# Surfing follicular waves in ovarian stimulation: is there a role for LH in DuoStim protocols? A narrative review and SWOT analysis

**DOI:** 10.1186/s12958-025-01360-9

**Published:** 2025-02-25

**Authors:** Alberto Vaiarelli, Danilo Cimadomo, Maria Cerrillo, Filippo Maria Ubaldi, Juan Antonio García Velasco

**Affiliations:** 1 IVIRMA Global Research Alliance, Genera, Clinica Valle Giulia, via G. de Notaris 2b, Rome, Italy; 2 IVIRMA Global Research Alliance, IVI, Madrid, Spain; 3 Department of Obstetrics and Gynecology, Rey Juan Carlos University, Madrid, Spain

**Keywords:** Follicular waves, DuoStim protocol, Unconventional ovarian stimulation, Luteinizing hormone, LH

## Abstract

IVF treatments should be personalized to collect an appropriate number of oocytes, taking into account the woman’s age and ovarian reserve, in order to maximize the efficacy and efficiency of the IVF process. From a scientific perspective, the ‘continuous recruitment theory’ suggests that several follicular waves are continuously recruited to grow and regress throughout one ovarian cycle. Clinically, this approach has paved the way for the theorization of a double stimulation protocol back-to-back in the same ovarian cycle (DuoStim) to rescue anovulatory waves. This protocol has been successfully adopted by several independent groups in the last decade to improve the number of oocytes in a short timeframe. Although the published data are promising for treating patients of advanced maternal age with reduced ovarian reserve and poor oocyte/embryo competence, the protocols adopted vary across studies. In this challenging population, choosing the appropriate protocol in the DuoStim context is critical to maximize the ovarian response and exploit the potential of individual follicular waves. In this regard, the administration of luteinizing hormone (LH) could be relevant to promote steroidogenesis and folliculogenesis, increase androgen production, improve pre-antral and antral follicle recruitment, and enhance the expression of follicle-stimulating hormone receptors in the granulosa cells. This review presents a step-by-step outline of all DuoStim protocols and proposes a SWOT (strengths, weaknesses, opportunities, threats) analysis of LH administration in the context of DuoStim.

**Supplementary Information:**

The online version contains supplementary material available at 10.1186/s12958-025-01360-9.

## Introduction

Follicular development within the ovary is a dynamic process: from a large pool of primordial follicles in the resting phase, some will develop as pre-antral and antral follicles until they undergo atresia, whereas others will complete their development after some further steps (sequential recruitment, selection, and follicle growth). According to the classic ‘single recruitment episode theory’, a single cohort of antral follicles grows during the follicular phase of the ovarian cycle after luteal regression [1]. However, this theory has been superseded by evidence of multiple waves arising during an ovarian cycle in many mammals. This evidence, first reported in large animal models, has been confirmed also in humans, leading to the definition of two further theories of follicle recruitment: ‘continuous recruitment theory’, according to which the follicles start growing and regress continuously during the ovarian cycle, and the ‘waves theory’, according to which two to three cohorts of antral follicles based on cycle length are recruited per ovarian cycle [1]. However, the mechanisms regulating each individual cohort of follicles are yet to be fully elucidated [2–5].

Several intraovarian regulators, follicle-stimulating hormone (FSH) and progesterone levels, and inflammatory markers (e.g., serum C-reactive protein) were all proposed as modulators of the dynamics behind the origin of follicular waves, but the molecular pathways describing their role remain poorly understood. From a clinical perspective, the growing knowledge of human ovarian follicular waves opened new options for ovarian stimulation (OS) to improve its efficacy and efficiency in specific patient populations undergoing in vitro fertilization (IVF):

- *Random start approach*: OS can be started at any time during the ovarian cycle (e.g., urgent fertility preservation).
- *Luteal phase stimulation*: OS can be started between day 17 and day 21 of a spontaneous ovarian cycle.
- *DuoStim* (double stimulation in a single ovarian cycle): the combination of two stimulations and two oocyte retrievals back-to-back in the same ovarian cycle [3].

Currently, cumulative live birth rate (CLBR) is the measure of IVF success [6–8]; however, the reproductive journey is as important as the destination. Improving IVF efficiency is equally critical, not only in terms of reduction of adverse events such as multiple pregnancy and ovarian hyperstimulation syndrome (OHSS), but also in shortening time to live birth, providing a cost-effective treatment, and minimizing dropout rates [9]. OS is key for success in assisted reproductive technology; therefore it is essential to select the proper protocol and the correct gonadotropin (Gn) type and starting dose, to avoid excessive response as well as hypo-response. In this regard, an accurate prediction of OS through the currently available markers of the ovarian reserve, namely anti-Müllerian hormone (AMH) and antral follicular count (AFC), is essential. However, a more thorough profiling of the patients requires the evaluation of at least age and body mass index, as well as experience of previous poor response [10].

Recently, genetic/genomic investigations of different reproductive phenotypes, pharmacogenomics, and molecular embryology might introduce potential tools to enhance OS management [11]. OS personalization can no longer be restricted to the choice of Gn starting dose. Indeed, it now involves many steps, including pre-treatment strategies, luteinizing hormone (LH) suppression regimens, types of Gn with LH or human chorionic gonadotropin (hCG) activity, and different types of trigger, as well as the possibility to use unconventional OS strategies [12]. Among the latter strategies, DuoStim represents the most intriguing, since by exploiting ovulatory and anovulatory follicular waves it may help poor-prognosis patients to optimize use of their ovarian reserve in the shortest possible timeframe. This narrative review provides a comprehensive overview of the various DuoStim protocols published over the years by different research groups, with a particular focus on analyzing the use of LH during hormonal stimulation.

### Search procedure

This narrative review was conducted by searching MEDLINE (PubMed), Scopus, and Embase databases up to December 2022. Combinations of the following keywords and search terms were used: ‘DuoStim’, ‘luteal phase stimulation’, ‘luteal phase ovarian stimulation’, ‘dual stimulation’, ‘double stimulation’, ‘ovarian stimulation’. Language restriction was adopted to select only papers in English. The reference lists of relevant reviews and articles in press were also hand-searched. Three reviewers (AV, DC, and MC) evaluated titles and abstracts. Disagreements were discussed and ultimately resolved by consensus between all authors also involving senior authors (FMU, JAGV). Twenty-four studies were considered eligible (Table 1).

**Table 1:** Summary of the DuoStim protocols adopted across all studies conducted to date in advanced maternal age ± poor ovarian responder patients. The references are in chronological order starting from the studies where LH was not administrated, followed by studies where it was administered either in the I or the II stimulation, and then by the studies where it was administered in both stimulations

Paper	Design	Number of patients	Maternal age	AFC	First OS				Second OS				Lab strategies
					Main Gn	LH administration	LH suppression	Trigger	Main Gn	LH administration	LH suppression	Trigger	
Kuang 2014 [13]	Pilot	38	36 y	3.8	CC 25 mg/d + LE 2.5 mg/d + HMG 150 IU/d	No	NSAID (ibuprofen) + PPOS (MPA)	GnRH-a	LE 2.5 mg/d + HMG 225 IU/d	No	PPOS (MPA)	GnRH-a	Fresh insemination + day 3 embryo vitrification
**Conclusion**: ↑ pre-ovulatory follicles and ↑ COCs in the second OS; No differences in maturation and fertilization rate between first and second OS													
Wei 2016 [14]	Retrospective	23	36 y	3	CC 50 mg/d or LE 2.5 mg/d + HMG 150 IU/d	No	PPOS (MPA)	GnRH-a	CC 50 mg/d or LE 2.5 mg/d + HMG 150 IU/d	No	PPOS (MPA)	hCG	Fresh insemination + day 3 embryo vitrification
**Conclusion**: ↑ COCs in the second OS; No differences in maturation and fertilization rate between first and second OS													
Zhang 2017 [15]	Retrospective	153	38 y	3	CC 50 mg/d + HP-FSH	No	NSAID (ibuprofen)	GnRH-a	HP-FSH	No	NSAID (ibuprofen)	GnRH-a	Fresh insemination + day 3 embryo vitrification
**Conclusion**: ↑ COCs and ↑ MII in the second OS													
Cardoso 2017 [16]	Retrospective	54	41 y	NR	rec-FSH 225 IU/d + HMG 75 IU/d	No	GnRH-ant	GnRH-a	rec-FSH 225 IU/d + HMG 75 IU/d	No	GnRH-ant	GnRH-a	Fresh insemination + blastocyst vitrification
**Conclusion**: No comparison between first and second OS													
Zhang 2018 [17]	Retrospective	61	39 y	5	CC 50–100 IU/d + HMG 75–150 IU/d	No	PPOS (dydrogesterone)	Rec-hCG	CC 50–100 IU/d + HMG 75–150 IU/d	No	PPOS (dydrogesterone)	Rec-hCG	Fresh insemination + day 3 embryo vitrification
**Conclusion**: ↑ COCs and ↓ maturation rate in the second OS													
Rashtian and Zhang 2018 [18]	Retrospective	69	42 y	5	CC 50 mg/d + LE 2.5 mg/d + rec-FSH 75 IU/d	No	GnRH-ant	GnRH-a	CC 50 mg/d + LE 2.5 mg/d + rec-FSH 75 IU/d	No	GnRH-ant	hCG	NR
**Conclusion**: No differences in pre-ovulatory follicles, COCs and maturation rate between first and second OS													
Madani 2018 [19]	Prospective	121	37 y	NR	CC 25 mg/d + LE 2.5 mg/d + HMG 150 IU/d	No	NSAID (ibuprofen) + PPOS (MPA)	GnRH-a	LE 2.5 mg/d + HMG 225 IU/d	No	PPOS (MPA)	GnRH-a	Fresh insemination + day2–3 vitrification
**Conclusion**: ↑ fertilization rate in the first OS. No differences in COCs between first and second OS													
Jin 2018 [20]	Retrospective	76	37 y	4	CC 50 mg/d + LE 5–10 mg/d + HMG 150–300 IU/d	No	GnRH-ant	GnRH-a	CC 50 mg/d + HMG 150–300 IU/d	No	GnRH-ant	hCG	Fresh insemination + day 3 embryo or blastocyst vitrification
**Conclusion**: ↑ COCs in the second OS. No differences in fertilization and maturation rate between first and second OS													
Hatirnaz 2019 [21]	Retrospective	51	29 y	1	rec-FSH 150–225 IU (single dose)	No	No	hCG	LE 5 mg/ day + rec-FSH 225 IU (single dose)	No	No	hCG	Fresh insemination + day 3 embryo vitrification
**Conclusion**: No comparison between first and second OS													
Eftekhar 2020 [22]	Cross-sectional study	10	31 y	> 5	rec-FSH 150 IU (single dose)	No	GnRH-ant	GnRH-a	HMG 300 IU/d	No	GnRH-ant	hCG	NR
**Conclusion**: ↑ COCs, MII and number of embryos in the second OS. No difference in embryo quality between the two groups													
Luo 2020 [23]	Retrospective	304	38 y	5	rec-FSH or HP-FSH 150–300 IU/d + HMG 150–300 IU/d	No	PPOS (MPA)	hCG or rec-hCG or GnRH-a	HMG 225 IU/d	No	PPOS (MPA)	hCG or rec-hCG or GnRH-a	Fresh insemination + day 3 embryo or blastocyst vitrification
**Conclusion**: ↑ pre-ovulatory follicles, COCs, maturation rate, and blastulation rate in the second OS													
Bourdon 2020 [24]	Retrospective	77	35 y	9	rec-FSH 300 IU/d or HMG 300 IU/d	No	GnRH-ant	GnRH-a	rec-FSH 300 IU/d or HMG 300 IU/d	No	GnRH-ant	GnRH-a	NR
**Conclusion**: ↓ COCs from the second stimulation													
Cecchino 2021 [25]	Retrospective	79	38 y	7	rec-FSH 200 IU/d ± HMG	No	GnRH-ant	GnRH-a or hCG	rec-FSH 200 IU/d ± HMG	No	GnRH-ant	GnRH-a or hCG	Fresh insemination + TE biopsy and CCT + blastocyst vitrification
**Conclusion**: ↑ COCs and MII in the second OS. No differences in maturation, fertilization, and blastulation rate between first and second OS													
Li 2022 [26]	Retrospective	89	34 y	6	CC 50–100 mg/d + HMG 225 IU/d	No	NSAID (indomethacin)	GnRH-a + hCG	CC 50–100 mg/d + HMG 225 IU/d	No	NSAID (indomethacin)	GnRH-a + hCG	Fresh insemination + day 3 embryo or blastocyst vitrification
**Conclusion**: No comparison between first and second OS													
Liu 2017 [27]	Retrospective	116	42 y	7	rec-FSH 150–300 IU/d	rec-LH 75–150 UI/d	GnRH long agonist or GnRH short agonist or GnRH-ant or PPOS (MPA)	Rec-hCG	HMG 225 IU/d	No	GnRH long agonist or GnRH short agonist or GnRH-ant or PPOS (MPA)	Rec-hCG	Fresh insemination + day 3 embryo or blastocyst vitrification
**Conclusion**: ↑ COCs, MII and fertilized oocytes in the second OS													
Lin 2018 [28]	Pilot	30	40 y	3	CC 100 IU/d + HMG 225 IU/d	No	GnRH-ant	GnRH-a + hCG	rec-FSH 300 IU/d	rec-LH 150 IU/d	PPOS (MPA)	GnRH-a + hCG	Fresh insemination + day 3 embryo vitrification
**Conclusion**: ↑ COCs, MII and fertilized oocytes in the second OS													
Ubaldi 2016 [29]	Retrospective	51	39 y	5	rec-FSH 300 IU/d	rec-LH 75 IU/d	GnRH-ant	GnRH-a	rec-FSH 300 IU/d	rec-LH 75 IU/d	GnRH-ant	GnRH-a	Fresh insemination + TE biopsy and CCT + blastocyst vitrification
**Conclusion**: No difference in euploid blastocyst rate per injected MII between first and second OS													
Cimadomo 2018 [30]	Retrospective	188	40 y	5	rec-FSH 300 IU/d	rec-LH 150 IU/d	GnRH-ant	GnRH-a	rec-FSH 300 IU/d	rec-LH 150 IU/d	GnRH-ant	GnRH-a	Fresh insemination + TE biopsy and CCT + blastocyst vitrification
**Conclusion**: ↑ COCs and MII oocytes in the second OS. No difference in fertilization, blastulation and euploidy rate between first and second OS													
Vaiarelli 2018 [31]	Retrospective	336	40 y	5	rec-FSH 300 IU/d	rec-LH 150 IU/d	GnRH-ant	GnRH-a	rec-FSH 300 IU/d	rec-LH 150 IU/d	GnRH-ant	GnRH-a	Fresh insemination + TE biopsy and CCT + blastocyst vitrification
**Conclusion**: ↑ COCs and fertilized oocytes in the second OS. No differences in fertilization, blastulation and euploid blastocyst rate between first and second OS													
Alsbjerg 2019 [20]	Case series	54	37 y	4	Corifollitropin alfa + rec-FSH 300–375 IU/d	rec-LH 150 IU/d	GnRH-ant	GnRH-a	Corifollitropin alfa + rec-FSH 300–375 IU/d	rec-LH 150 IU/d	GnRH-ant	hCG	Fresh insemination + day 3 or blastocyst vitrification
**Conclusion**: ↑ COCs in the second OS													
Vaiarelli 2020 [3]	Multicenter, observational study	827	39 y	< 6	rec-FSH 300 IU/d	rec-LH 150 IU/d	GnRH-ant	GnRH-a	rec-FSH 300 IU/d	rec-LH 150 IU/d	GnRH-ant	GnRH-a	Fresh insemination + TE biopsy and CCT + blastocyst vitrification
**Conclusion**: ↑ COCs and MIIs in the second OS. No differences in clinical, obstetric, and perinatal outcome after euploid blastocysts obtained from first or second stimulation													
Vaiarelli 2020 [32]	Retrospective	100	42 y	3	rec-FSH 300 IU/d	rec-LH 150 IU/d	GnRH-ant	GnRH-a	rec-FSH 300 IU/d	rec-LH 150 IU/d	GnRH-ant	GnRH-a	Fresh insemination + TE biopsy and CCT + blastocyst vitrification
**Conclusion**: ↑ COCs and MIIs in the second OS. ↓ Cycle discontinuation after the first failed attempts in DuoStim vs. two conventional strategies													
Cerrillo 2022 [33]	RCT	41	39 y	6	HMG 225–300 IU/d or rec-FSH 225–300 IU/d	rec-LH 112.5–150 IU/d *(in case of rec-FSH)*	GnRH-ant	GnRH-a	HMG 225–300 IU/d or rec-FSH 225–300 IU/d	rec-LH 112.5–150 IU/d *(in case of rec-FSH)*	GnRH-ant	GnRH-a	Vitrified-warmed (I stim) and fresh (II stim) insemination + TE biopsy and CCT + blastocyst vitrification
**Conclusion**: ↓ time to obtain an euploid embryo. ↑ fertilization rate in the second OS. No differences in COCs and MIIs between first and second OS													
Vaiarelli 2022 [34]	Retrospective	143	41 y	NR	rec-FSH 300 IU/d	rec-LH 150 IU/d	GnRH-ant	GnRH-a	rec-FSH 300 IU/d	rec-LH 150 IU/d	GnRH-ant	GnRH-a	Fresh insemination + TE biopsy and CCT + blastocyst vitrification
**Conclusion**: ↓ Cycle discontinuation after the first failed attempts and ↓ time between the first and the second OS compared with two conventional OS													

*AFC *antral follicle count, *CC *clomiphene citrate, *COC *cumulus cell-oocyte complex, *Gn *gonadotropin, *GnRH-a *GnRH agonist, *hCG *human chorionic gonadotropin, *HMG *human menopausal gonadotropin, *HP-FSH *highly purified follicle stimulating hormone, *LE *letrozole, *LH *luteinizing hormone, *MII *metaphase II oocytes, *MPA *medroxyprogesterone acetate, *NR *not reported, *NSAID *non-steroidal anti-inflammatory drug, *OS *ovarian stimulation, *PPOS *progestin primed ovarian stimulation, *rec-FSH *recombinant follicle stimulating hormone, *rec-LH *recombinant luteinizing hormone, *r-HCG *recombinant human chorionic gonadotropin

### DuoStim: the optimal framework

**Fig. 1:**
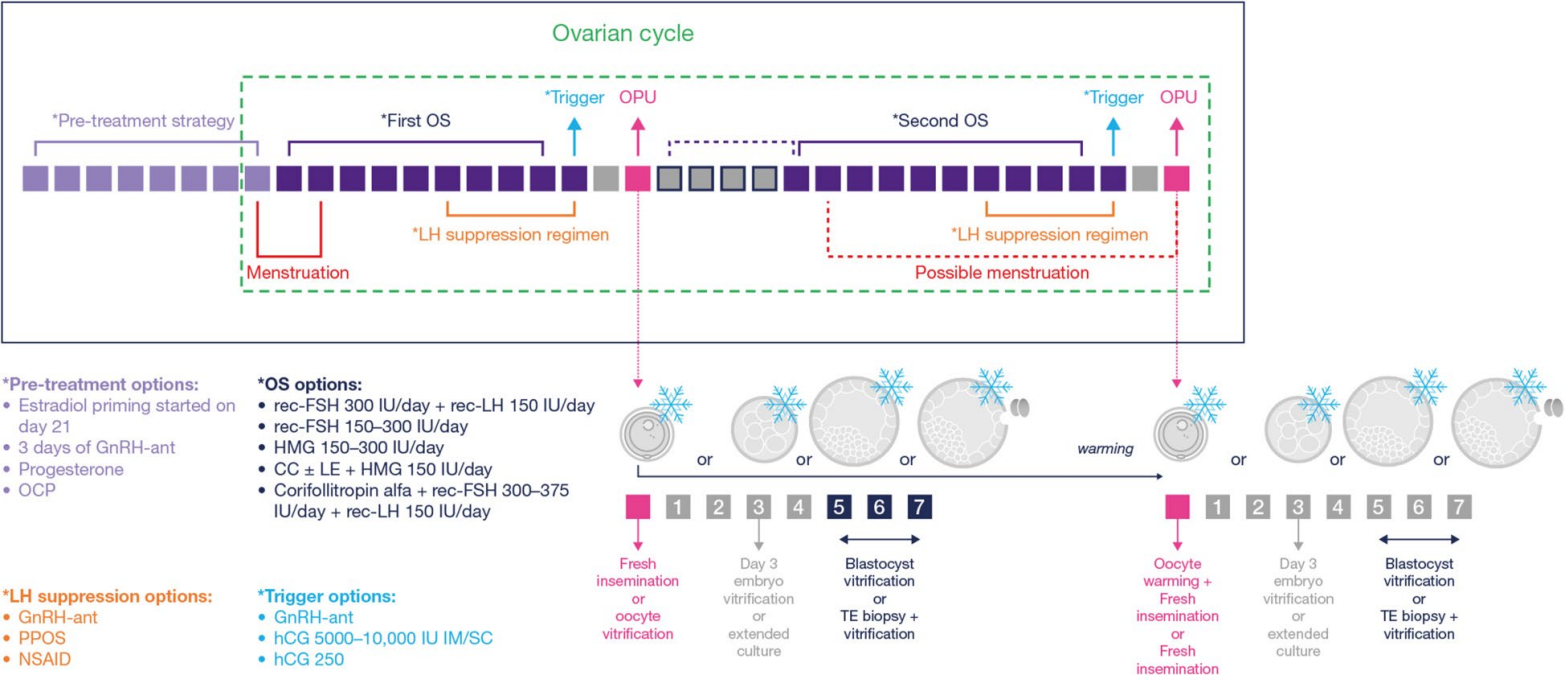
Framework of DuoStim protocol. Each square represents a day. Violet squares identify the days before the beginning of the first ovarian stimulation (OS), when pre-treatment strategies might be adopted. Purple squares represent the days of first and second OS. Gray squares represent the days when no treatment is applied; if their contour is purple, they identify the possibility of starting the second OS soon after the first retrieval, as for the Shanghai protocol. Dark pink squares represent the days of oocyte pick-up (OPU). Light blue arrows identify ovulation trigger administration. Red brackets identify the timeframe when (possible) menstruation might occur. Orange brackets identify the timeframe of LH suppression regimen administration. The ovarian cycle is framed within a dotted green larger square. All possible pre-treatment, OS, LH suppression, and trigger options are listed in the figure. In the lower right corner, all possible lab strategies are shown: fresh oocytes insemination after both OPUs or oocyte cryopreservation after the first OPU, then warmed and inseminated along with the fresh oocytes obtained from the second OPU (dotted dark blue arrow) plus cleavage-stage embryo cryopreservation or blastocyst culture and blastocyst cryopreservation with/without trophectoderm (TE) biopsy for pre-implantation genetic testing. The squares mirror the same squares in the DuoStim protocol framework. The white numbers within each square are the days after OPU. GnRH-ant, GnRH antagonist; OCP, oral contraceptive pill; HMG, human menopausal gonadotropin; CC, clomiphene citrate; LE, letrozole; PPOS, progestin-primed ovarian stimulation; NSAID, non-steroidal anti-inflammatory drug

Several DuoStim protocols have been proposed to date (Table 1; Fig. 1) (e.g [14–22, 24–28, 32, 35, 36]). , , but hard data are missing to support the superiority of a specific protocol over others [23]. Independent studies worldwide outlined consistently good and reproducible results in terms of (i) more mature oocytes obtained, and more embryos available in a single ovarian cycle [37]; (ii) lower dropout rates between consecutive failed attempts in poor-prognosis patients [3]; and (iii) similar outcomes as double conventional stimulation but potentially increased flexibility and patient compliance [38]. All steps of DuoStim protocol are summarized in Fig. 1 and detailed in the next paragraphs.

### Estradiol priming started in day 21 of the previous menstrual cycle

Luteal estradiol priming (4 mg/d of estradiol valerate) can be adopted in day 21 of the previous menstrual cycle [29, 39]. In fact, pre-treatment therapies – not only estrogen priming, but also administration of progesterone, oral contraceptive pill (OCP), or gonadotropin-releasing hormone (GnRH) antagonists – have all been proposed in conventional OS to suppress or reduce LH and/or FSH secretion. They can be administered before OS with the aim of (i) synchronizing follicular development; (ii) preventing early large follicle or spontaneous LH surge; (iii) reducing cyst formation; and (iv) scheduling IVF to improve cycle management and workflow. Future studies must confirm the utility of these pre-treatment strategies in the DuoStim context.

### Gonadotropin type and daily dose

Many regimens with different Gn type and dosage have been proposed in the literature to optimize the ovarian response in terms of number of oocytes retrieved and oocyte/embryo quality during either conventional OS or DuoStim. Clearly, the number of oocytes is strongly associated with an improved CLBR per cycle and maximizing ovarian response to OS is critical. However, the real value of different stimulation protocols to enhance the ovarian response is still a matter of debate, especially in terms of follicle recruitment and oocyte quality. In the DuoStim context, different protocols have been proposed [3, 23, 38, 40], such as:

- Clomiphene citrate (CC) 50–100 mg/d and/or letrozole 2.5 mg + human menopausal gonadotropin (HMG) 150–300 IU/d.
- Recombinant (rec)-FSH or HMG 150–300 IU/d.
- Corifollitropin alfa + rec-FSH 300–375 IU.
- Rec-FSH 300 IU/d + rec-LH 75–150 IU/d.

However, based on the current body of evidence in conventional stimulations, the true impact of Gn remains an open question. In this regard, for a deeper evaluation of the efficacy of different types of Gn, two aspects should be considered: (i) ovarian sensitivity, defined as Follicular Output RaTe (FORT) [41] and Follicle to Oocyte Index (FOI) [42]; and (ii) embryo competence, in terms of blastocyst development, euploidy, and implantation [12, 43–45]. Although future studies are still needed to analyze the effect of OS on these parameters, mounting evidence supports no impact imputable to Gn type or dose, number of oocytes retrieved, or OS duration [12, 46–48]. In this scenario, maximizing the ovarian response, rather than adopting a mild stimulation approach, is key especially in poor-prognosis patients, to enhance the CLBR while minimizing the risk for cycle cancellation [49]. Some data suggest that OS with rec-FSH may allow the retrieval of larger cohorts of oocytes with respect to HMG alone. Additional putative benefits of rec-FSH are higher patient compliance (because of lower Gn dose, shorter OS, and easier route of administration) and cost-effectiveness [50–53]. For all these reasons, rec-FSH might be considered the most suitable molecule in the context of DuoStim.

### LH administration to enhance ovarian response in DuoStim

**Fig. 2:**
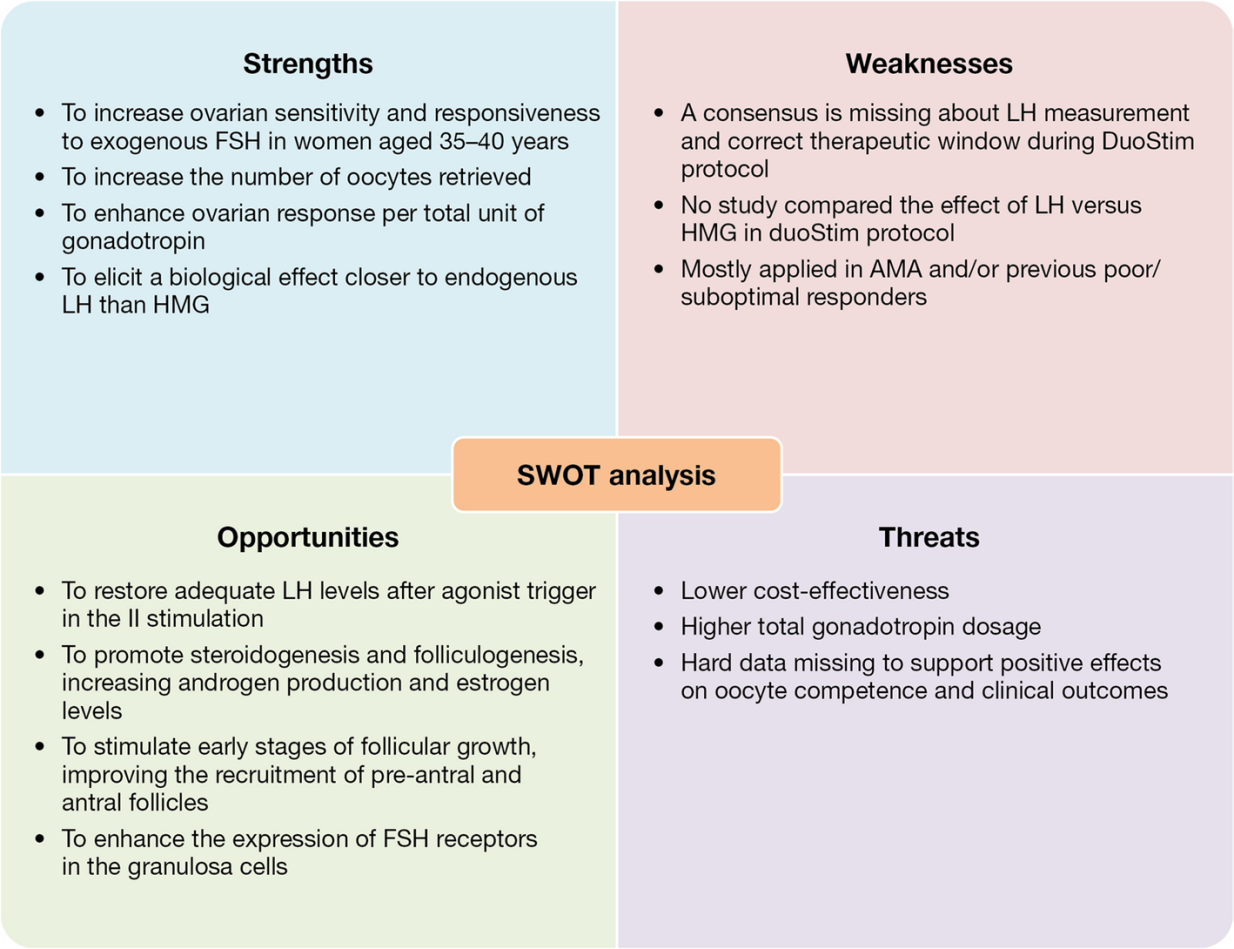
SWOT analysis of LH administration in the context of DuoStim protocol in advanced maternal age and/or previous poor/suboptimal responders

LH administration during unconventional OS, including DuoStim, is still an unexplored topic. In fact, to date, the rationale guiding the use of this molecule is based on evidence from studies with conventional OS approaches. Specifically, the choice of adding rec-LH in DuoStim protocols is based on the low androgen levels characterizing most poor-prognosis patients. Rec-LH could promote steroidogenesis and folliculogenesis increasing androgen production, improving pre-antral and antral follicle recruitment, and increases the expression of FSH receptors in the granulosa cells [54–57]. All these aspects are crucial in advanced maternal age and/or poor/suboptimal responder patients, whose decreased androgen levels may further impact ovarian sensitivity and responsiveness to exogenous FSH [58]. On this basis, rec-LH co-treatment during OS could be adopted in subgroups of poor-prognosis women, such as women aged 35–40 years [58] and hypo-responders [59]. Although the clinical value of LH administration is still debated and a consensus is missing on its measurement and adequate therapeutic window, reports exist in poor-prognosis patients supporting lower rec-FSH dose and better IVF outcomes, with no increased costs, when it is supplemented during OS [60, 61]. These data overall support its adoption in the DuoStim protocol. A SWOT analysis (Fig. 2) was included in this review to summarize the ‘strengths, weaknesses, opportunities, and threats’ of LH adoption in DuoStim protocols, based on the current clinical and academic body of evidence.

### LH suppression regimens

LH suppression regimens involve GnRH agonists or antagonist administration to avoid LH surge, thereby preventing premature ovulation during OS [62]. Over time, antagonist protocols gained more popularity in OS due to their numerous advantages, such as (i) shorter overall treatment duration, (ii) lower Gn consumption, (iii) absence of perimenopausal symptoms caused by pituitary desensitization, and (iv) use of agonist trigger to either minimize OHSS risk or allow a second stimulation in the same ovarian cycle (DuoStim). Indeed, agonist trigger is commonly adopted during DuoStim since it reduces corpora lutea half-life, thus allowing an optimal hormonal environment for the administration of exogeneous Gn in the same ovarian cycle. This additional Gn administration will ultimately support the final maturation of follicles that would otherwise undergo atresia. More recently, the increasing evidence supporting progestins to inhibit spontaneous ovulation during OS without compromising the ovarian response opens a new era in the management of unconventional protocols. Indeed, progestins might represent a reliable and more convenient tool to replace GnRH antagonist administration, thus reducing the number of monitoring visits and the overall cost of OS [63]. More studies are needed to evaluate this strategy.

### Type of trigger

The trigger for final oocyte maturation is administered about 35–36 h before oocyte retrieval and it is critical for the re-initiation and completion of the first meiotic division as well as for oocyte cytoplasmic maturation. The timing of its administration during OS is based on accumulated information about follicles, hormonal data, OS duration, and ovarian response to OS. hCG trigger is considered the gold standard in conventional IVF where fresh transfers are scheduled. However, GnRH-agonist trigger, by stimulating the pituitary gland to secrete both endogenous LH and FSH, represents the first-line choice in freeze-all cycles, such as among patients showing high response to OS [64], donors, and in fertility preservation [65, 66], as well as in DuoStim which is commonly used [13]. The absence of an impact of the trigger, either hCG or GnRH agonist, on oocyte competence is supported by at least three studies conducted in the pre-implantation genetic testing for aneuploidies (PGT-A) setting at the blastocyst stage with comprehensive chromosome testing technologies [67–69].

The most important advantage of GnRH-agonist trigger in the context of DuoStim is its shorter half-life, which reduces the permanence of corpora lutea and the length of the luteal phase. In this regard, although luteolysis is patient-specific and highly dependent on hormonal levels, the number of oocytes retrieved and the number of corpora lutea [70], the administration of GnRH agonist trigger is ideal to allow re-starting a II stimulation in the same ovarian cycle [31]. On a separate note, a possible flare-up effect may derive from GnRH agonist trigger in the I stimulation, which might induce a down-regulation in AMH expression in the follicles from the anovulatory wave, thereby increasing the number of follicles with 3–4 mm diameter potentially recruitable during the II stimulation [71].

### Second ovarian stimulation in the same ovarian cycle: starting day

In the Shanghai protocol, the II stimulation was commenced the day after first oocyte retrieval, when two or more antral follicles were identified. However, this strategy is subject to errors because the evaluation of the antral follicles in the follicular waves arising in the luteal phase is compromised by the presence corpora lutea from the first retrieval. A simpler approach was defined by Ubaldi and colleagues, who preferred to start the second stimulation after 5 days from the first retrieval, namely when complete luteolysis is attained [29]. In this workflow, II stimulation is conducted with the same protocol and daily dose as the I stimulation, regardless of the number of antral follicles. Moreover, when oocytes are not vitrified after the I stimulation, it can be decided even in progress whether or not to start a II stimulation, based on maternal age and the number of blastocysts available for biopsy in PGT cycles obtained. This allows also a more patient-centered and personalized treatment [34].

### Biological strategies in the context of DuoStim

An efficient oocyte and embryo cryopreservation program is key in modern IVF. As with any unconventional OS approach, DuoStim could not be applied in the absence of this critical prerequisite, due to the asynchrony between follicular development and endometrial cycles. When optimized in each clinic, cryopreservation allows oocyte accumulation strategies to counteract the effect of aging on both a diminished ovarian reserve and poor oocyte quality [72], even though no impact on euploidy rates at the blastocyst stage has been reported [73, 74]. A putative impact on blastocyst development might derived from oocyte vitrification [73, 74], but evidence for this is controversial [75–77]. It is therefore still a matter of discussion which strategy – oocyte vitrification, or embryo vitrification (if allowed by local regulations) – is preferable after the I stimulation. When dealing with very advanced maternal age women (especially in their 40s), namely those with a clear indication to DuoStim, aneuploidy testing to report non-mosaic aneuploidies is desirable to reduce the risk of miscarriage, while increasing our prediction upon embryo competence.

In summary, the implementation of DuoStim strategy is secondary to the achievement of high standards in the IVF lab, which should be testified by competency, if not benchmark, values across all the main key performance indicators outlined by international scientific societies [78], including for trophectoderm biopsy [79].

### SWOT analysis regarding the role of LH during the DuoStim protocol

To summarize the potential advantages and disadvantages of DuoStim in women of advanced maternal age and/or those who have had a poor/suboptimal response to conventional approaches, a SWOT analysis was conducted (Fig. 2). This analytical framework is useful for summarizing the strengths, weaknesses, opportunities, and threats of this strategy. The strengths of this approach include increased ovarian sensitivity and responsiveness to exogenous FSH in women aged 35–40, resulting in a larger number of retrieved oocytes. Additionally, there is an enhancement of ovarian response per total unit of gonadotropin and a biological effect that is more similar to endogenous LH than HMG effect. Weaknesses in the DuoStim protocol include the absence of a consensus on LH measurement and its therapeutic window, the lack of studies comparing the effects of LH versus HMG, and the protocol’s limited application to advanced maternal age and/or previous poor/suboptimal responders. The aim is to restore adequate LH levels after agonist trigger in the second stimulation, promote steroidogenesis and folliculogenesis by increasing androgen production and estrogen levels, stimulate early stages of follicular growth to improve the recruitment of pre-antral and antral follicles, and enhance the expression of FSH receptors in the granulosa cells. The potential drawbacks of this approach include the absence of a cost-effectiveness analysis, the administration of high doses of gonadotrophins, and the lack of concrete evidence supporting positive effects on oocyte competence and clinical outcomes. In order to address these weaknesses, further studies are required to fully understand the potential risks and benefits of this method.

## Conclusions

Evidence of multiple waves of follicular growth during one ovarian cycle has led to the development of novel ovarian stimulation strategies that aim to better exploit the ovarian reserve. The successful application of the random start protocol in the context of urgent fertility preservation or luteal phase stimulation for poor responders has led to the theorization of DuoStim. This approach offers a unique opportunity to increase the number of oocytes retrieved and embryos obtained within a shorter timeframe for a specific group of patients who require a higher quantity of gametes to achieve a live birth.

Independent studies worldwide have provided growing evidence that oocytes obtained from unconventional stimulations demonstrate the same competence in terms of fertilization, blastulation, and euploidy rates as those obtained from conventional stimulation. Additionally, euploid blastocysts from the II stimulation have been shown to have the same clinical, obstetric, and perinatal outcomes as those from the I stimulation. The current studies are insufficient to recommend an ideal gonadotropin protocol for this strategy. It is important to note that this information is not conclusive and further research is needed. However, using LH during stimulation may enhance ovarian response in DuoStim patients. The type of LH supplementation – LH versus hMG – is still a topic of debate and controversy among practitioners, which can cause confusion.

From a psychological perspective, DuoStim can be considered a perfect fit for a multicycle approach, as it involves multiple ovarian stimulations within a single therapeutic protocol. This approach is promising for specific patient populations who are candidates for IVF but have a low chance of obtaining a competent embryo. By accounting for two stimulations, DuoStim approach inherently involves the discussion with the couple that treatment unsuccess is a possibility and allows an upfront discussion of the benefits of multiple retrievals. Perhaps, two conventional stimulations in two consecutive cycles could achieve comparable results, but also entail a high risk of treatment discontinuation and longer time to obtain a euploid blastocyst and, ultimately, a live birth. Further studies are needed to investigate this approach, both in the context of randomized trials and real-life experiences.

## Supplementary Information

Additional file 1.

## Data Availability

Not applicable.

## Abbreviations

AFC: Antral follicular count
AMH: Anti-Müllerian hormone
CC: Clomiphene citrate
CLBR: Cumulative live birth rate
FOI: Follicle to Oocyte Index
FORT: Follicular Output Rate
FSH: Follicle-stimulating hormone
Gn: Gonadotropin
GnRH: Gonadotropin-releasing hormone
hCG: Human chorionic gonadotropin
HMG: Human menopausal gonadotropin
IVF: In vitro fertilization
LH: luteinizing hormone
NSAID: Non-steroidal anti-inflammatory drug
OCP: Oral contraceptive pill
OHSS: Ovarian hyperstimulation syndrome
OPU: Oocyte pick-up
OS: Ovarian stimulation
PGT: Pre-implantation genetic testing
SWOT: Strengths, weaknesses, opportunities, and threats
