# Supplementary material for: Surfing follicular waves in ovarian stimulation: is there a role for LH in DuoStim protocols? A narrative review and SWOT analysis
Source: Reprod Biol Endocrinol. 2025 Feb 25;23(Suppl 1):28. doi: 10.1186/s12958-025-01360-9 (PMC11852546; doi:10.1186/s12958-025-01360-9)
Supplement: Supplementary file 1 — Additional file 1. [file 12958_2025_1360_MOESM1_ESM.docx]

# SUPP-D-23-00073: RESPONSES TO REVIEWERS

Surfing follicular waves in ovarian stimulation: Is there a role for LH in DuoStim protocols?

We sincerely thank the editor and reviewer for their supportive comments, which certainly improved the manuscript. Our detailed responses are shown below.

## Editor comments

| **Editor comment** | **Author response** |
| --- | --- |
| The authors are commended for providing a clear and concise overview about the DuoStim protocol.  The reviewer has also found merit in the paper, but provided constructive criticisms, to which I concur, and that I invite the authors to address.  In particular, data on outcomes and a more detailed discussion of the SWOT framework are crucial to enhance the paper's appeal. | Thanks for your comments. |

## Reviewer comments

I've read with interest the review article by Vaiarelli and co-authors on Duostim protocols for ovarian stimulation in the context of IVF.

The article is well-written yet concise, providing the most important data published on the matter concerned. Authors are commended for putting together such an elegant review.

I would take this opportunity to suggest some changes and ask the authors to provide additional clarifications as below.

| **Review comment** | **Author response** | **MS page #** | **MS line #** |
| --- | --- | --- | --- |
| **Abstract**: Too vague. Please include some of the key findings in the abstract. | We modified the abstract as suggested, trying to emphasize the content of the work and the goal of this paper. | 2 | 14–30 |
| **Introduction and Discussion**: Key statements lack citations (E.g., Introduction: 1st, 2nd, 3rd and 4th paragraphs). The definition of CLBR lacks citation (suggest using the one provided by the ICMART which refers to cumulative delivery rate). Please revise accordingly. | Thanks for your suggestion, we have integrated the following references in the introduction: PMID: 22068695, PMID: 10952925, PMID: 32338123, PMID: 12606405, PMID: 21411253, PMID: 31701304, PMID: 26724797, PMID: 38174816, PMID: 33197264, PMID: 36586005 | 3 | 34–79 |
| **Introduction**: Include an addition paragraph informing the reader about the scope of the review. Since its narrative, it's important to state how the information was collected to develop it. | Thanks for your suggestion, we have added an additional paragraph entitled “Search procedure” in which we explained how we selected the papers included in this narrative review. | 4–5 | 80–88 |
| Table and Figure are nicely presented and are appreciated. | Thanks. | – | – |
| Authors are requested to introduce the SWOT concept and briefly discuss each of its areas as a separate paragraph in the manuscript. | Thanks for your suggestion. We have created a new paragraph to discuss the pros and cons of using LH in Duostim protocols through a SWOT analysis. | 9–10 | 213–231 |
| Although the Table summarizes the key data of published evidence, no outcome data is provided. Authors are requested to elaborate on this by including an extra Table or incorporating outcome data in the respective sections | We have improved the table thanks to your observations by adding conclusions for each study. Additionally, we have included three more studies (below) in the table. We believe that this makes it easier to get an idea of the diversity of protocols used for this type of approach.   - Eftekhar M, Mohammadi B, Khani P, et al. Dual stimulation in unexpected poor responder POSEIDON classification group 1, sub-group 2a: A cross-sectional study. PMID: 32754681 PMCID: PMC7340986 DOI: 10.18502/ijrm.v13i6.7287 - Bourdon M, Santulli P, Maignien C, et al. The ovarian response after follicular versus luteal phase stimulation with a double stimulation strategy. PMID: 32046382 DOI: 10.1007/s43032-019-00012-9 - Vaiarelli A, Cimadomo D, Alviggi E, et al. The euploid blastocysts obtained after luteal phase stimulation show the same clinical, obstetric and perinatal outcomes as follicular phase stimulation-derived ones: a multicenter study. Hum Reprod. 2020 Nov 1;35(11):2598-2608. doi: 10.1093/humrep/deaa203.PMID: 32951051 | Table supplied as a separate file | |
| Please define 'very low prognosis patients' as indicated in the abstract. Readers would benefit to understand which patients might be eligible to undergo Duostim. | We have modified the abstract as requested and we have better encoded the target population for this approach, which could benefit from the use of LH during ovarian stimulation. | 2 | 14–30 |
